# Supplementary material for: Thromboembolic Events in Users of Warfarin Treated with Different Skeletal Muscle Relaxants
Source: Medicina (Kaunas). 2022 Aug 29;58(9):1171. doi: 10.3390/medicina58091171 (PMC9501796; doi:10.3390/medicina58091171)

SUPPLEMENTAL MATERIAL | eMethods

1. Study cohorts

Separate cohorts of adult users of warfarin (the object drug[10] of interest, the affected agent in a drug interaction pair) and inhaled corticosteroids (ICSs, the prespecified negative control object drug[10]) served as bases for the first eight and second eight self-controlled series studies (SCCSs), respectively. We utilized National Drug Codes and days' supply values on prescription dispensing claims to construct object drug exposure episodes. We permitted a grace period (**eMethods Table 1**) between contiguous object drug dispensings (and at the end of the terminal object drug dispensing) to account for imperfect adherence. We repeated this approach for dispensings of muscle relaxants (precipitant drugs,[10] affecting agents in a drug interaction pair). Among object drug users, we therefore identified periods with and without exposure to muscle relaxants. We then limited each cohort to persons with an outcome during their observation period (defined below), a requirement of the case-only SCCS design. If fewer than 10 persons constituted a given cohort, we excluded it from further study.

eMethods Table 1. Approach to defining grace period length

Operational Definition	Examples	Rationale
We permitted the grace period length to vary based on the days' supply (DS) of the prescription claim. Length was calculated as (DS x 0.2), rounded-up to the nearest integer. We set a minimum grace period length of one day.	For a warfarin prescription dispensed in a mail order pharmacy setting, we would expect a median DS = 90 days. Therefore, the grace period length would be (90 x 0.2) = 18 days. For a cyclobenzaprine prescription dispensed in a retail pharmacy setting, we would expect a median DS = 30 days. Therefore, the grace period length would be (30 x 0.2) = 6 days.	Proportional measure to permit suboptimal adherence

We prespecified ICSs as negative control object drugs since these agents: a) are used chronically, like warfarin; b) have no known interactions with muscle relaxants;[18] and c) are not themselves independently associated with thromboembolism.[19] The following ICSs, with a labeled indication for asthma, were included for study: beclomethasone; budesonide; ciclesonide; flunisolide; fluticasone; and mometasone.

2. Observation and pre-observation baseline periods

For each individual meeting inclusion criteria, the observation period began with object drug initiation and was censored upon the earliest of: a) lapsed exposure to the object, defined by exhausting days' supply (plus terminal grace period); b) switching to a therapeutic alternative (e.g., dabigatran in a warfarin user, methylprednisolone in an ICS user); c)

Medicaid disenrollment; d) the end of the study dataset; and e) death. We did not censor observation time upon outcome occurrence since this would violate a tenet of the SCCS design.[20]

The 183 days immediately preceding the observation period served as a baseline period. We required it to be devoid of a/an: a) dispensing for the object drug or therapeutic alternative; and b) interruption in Medicaid coverage. The former criterion served to restrict the study to incident users of the object drug, thereby eliminating prevalent user bias and minimizing the likelihood of overadjustment bias.[21]

3. Observation period follow-up time

We dichotomized each day of the observation period as an exposed or unexposed day, based on the presence or absence of exposure to a muscle relaxant. Consistent with standard practice[20] and to minimize exposure trend bias,[22] we employed a bidirectional implementation of the SCCS design that permitted for study unexposed days occurring before and after exposed days. Of note, each observation period was not required to have exposed and unexposed days, yet only such instances contributed to the estimation of incidence rate ratios (IRRs) for the association between muscle relaxants drug exposure and outcome. See **eMethods Figure 1** for a graphical representation of observations eligible for inclusion.

eMethods Figure 1. Examples of observation periods eligible for inclusion; P-U = precipitant unexposed; P-E = precipitant exposed; VTE = venous thromboembolism

Panel A: Individual with P-E and P-U person-days of observation

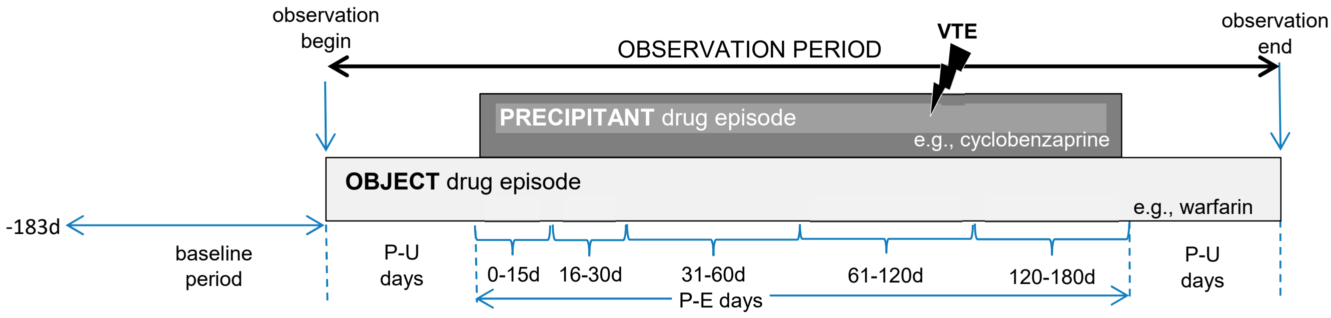

Panel B: Individual solely with P-E person-days of observation

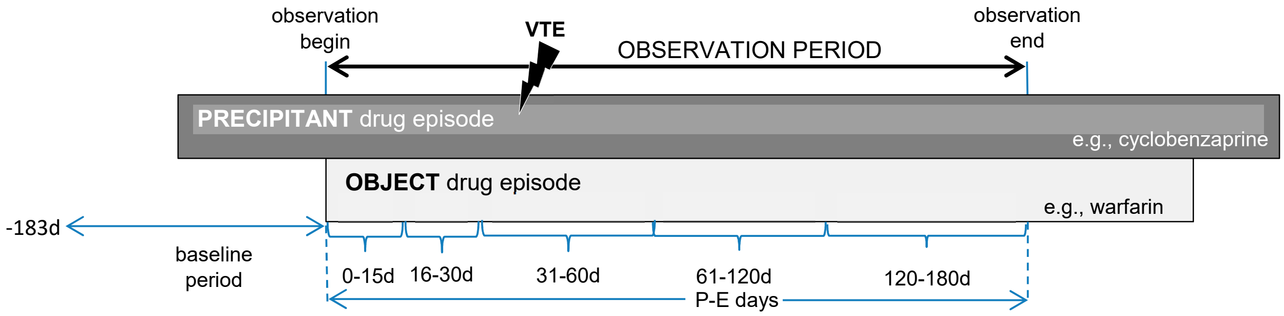

Prior studies indicate that adverse events attributable to a pharmacokinetic drug interaction peak shortly after initiating concomitant therapy.[23-25] We therefore conducted duration-response analyses to elucidate time-varying hazards.[10] We stratified exposed time into the following mutually exclusive periods from initiation of concomitance: 0–15, 16–30, 31–60, 61–120, and 121–180 days.

4. Exposure of interest and covariates

The exposure of interest was the muscle relaxant precipitant drug initially defining concomitance with the object drug. Precipitants included baclofen, carisoprodol, chlorzoxazone, cyclobenzaprine, metaxalone, methocarbamol, orphenadrine, and tizanidine. Consistent with convention, we considered baclofen to be an antispastic agent, tizanidine to be an antispastic-antispasmodic agent, and the remaining muscle relaxants to be antispasmodic agents. We investigated these muscle relaxants in separate SCCSs with each object drug (i.e., eight precipitants x two object drugs = 16 studies).

The SCCS design innately controls for time-invariant covariates.[27] We therefore considered only time-varying covariates as potential confounders. We included, in each regression model, covariates from the following categories: a) age; b) non-chronic risk factors for venous thromboembolism (VTE) or ischemic stroke (IS); c) drugs that can increase the risk of VTE, IS, or both;[28] d) non-chronic diseases that can affect coagulation; e) drugs that can affect coagulation; f) drugs that can affect cytochrome P450 hepatic metabolism;[29,30] g) non-chronic factors that portend skeletal muscle relaxant exposure; h) prior use of a skeletal muscle relaxant; and i) long-term care residence. For the warfarin cohorts, we also included the following covariates: j) therapeutic drug monitoring for warfarin; and k) average daily warfarin dose; in a secondary analysis we included average daily muscle relaxant dose. See **eMethods Table 2** for additional detail on included covariates.

eMethods Table 2. Time-varying covariates included in conditional Poisson regression models

Covariate, measured on each day of observation	Detail	Identification method	Inclusion in object drug cohort	
			warfarin	ICS
Follow-up month				
month	month of the current day of observation, as categorical variable [1 (ref), 2, 3, 4, …, 24+ months]	date	x	x
Non-chronic risk factors for thromboembolism				
hospital discharge	on current day of observation or in the 30 days prior	hospitalization claims	x	x
VTE	in the 90 days prior to the current day of observation	ICD-9-CM diagnosis codes	x	x
IS			x	x
Drugs that can increase the risk of thromboembolism				
oral contraceptives / HRT	on current day of observation or in the 30 days prior	NDCs	x	x
NSAIDs			x	x
tamoxifen			x	x
nicotine			x	x
SSRIs / SNRIs			x	x
testosterone			x	x
dexamethasone			x	x
methylprednisolone			x	x
flutamide			x	x
anastrozole			x	x
megestrol			x	x
estramustine			x	x
imatinib			x	x
lenalidomide			x	x
thalidomide			x	x
pentosan			x	x
clozapine			x	x
quetiapine			x	x
thioridazine			x	x
celecoxib			x	x
topiramate			x	x
recombinant factor VIIa		NDCs, HCPCSs	x	x
cisplatin			x	x
epoetin alfa / darbepoetin alfa			x	x
G-CSF / GM-CSF			x	x
goserelin			x	x
leuprolide			x	x
cyclosporine			x	x
infliximab			x	x
immune globulins			x	x
interferon gamma-1b			x	x
sirolimus / tacrolimus			x	x
aldesleukin			x	x
bevacizumab			x	x
bleomycin			x	x
carboplatin			x	x
denileukin			x	x
docetaxel			x	x
fluorouracil			x	x
irinotecan			x	x

paclitaxel			x	x
heparins, including LMWHs			x	x
chlorpromazine			x	x
olanzapine			x	x
risperidone			x	x
botulinum toxins			x	x
papaverine			x	x
Non-chronic diseases that can affect coagulation				
Infection, acute	on current day of observation or in the 14 days prior	ICD-9-CM diagnosis codes	x	x
Drugs that can affect coagulation				
anticoagulant, oral, non-warfarin	on current day of observation or in the 30 days prior	NDCs	x	
anticoagulant, oral				x
antiplatelet, oral			x	x
aspirin			x	x
anticoagulant, subcutaneous / injectable		NDCs, HCPCSs	x	x
Drugs that can interact*, e.g., affect CYP hepatic metabolism				
oral agents that can interact with warfarin, per Truven Health Analytics Micromedex Solutions	on current day of observation or in the 30 days prior (14 days prior for antibiotics)	NDCs	x	
oral agents that can interact with a skeletal muscle relaxant, per Truven Health Analytics Micromedex Solutions			x	x
CYP2C9 inhibitors, clinically relevant, oral			x	x
CYP2C9 inducers, clinically relevant, oral			x	x
CYP1A2 inhibitors, clinically relevant, oral			x	x
CYP1A2 inducers, clinically relevant, oral			x	x
CYP2C19 inhibitors, clinically relevant, oral			x	x
CYP2C19 inducers, clinically relevant, oral			x	x
CYP2D6 inhibitors, clinically relevant, oral			x	x
CYP2E1 inhibitors, clinically relevant, oral			x	x
CYP2E1 inducers, clinically relevant, oral			x	x
CYP3A4 inhibitors, clinically relevant, oral			x	x
CYP3A4 inducers, clinically relevant, oral			x	x
CYP2C8 inhibitors, clinically relevant, oral			x	x
CYP2B6 inhibitors, clinically relevant, oral			x	x
CYP2B6 inducers, clinically relevant, oral			x	x
Non-chronic factors that portend skeletal muscle relaxant exposure				
diseases of esophagus, including GERD	on current day of observation or in the 30 days prior	ICD-9-CM diagnosis codes	x	x

disorders of musculoskeletal system and connective tissue			x	x
hereditary and degenerative diseases of the CNS, pain, and other headache syndromes			x	x
injury			x	x
pain, jaw			x	x
pain, generalized			x	x
symptoms involving nervous and musculoskeletal systems, including tetany			x	x
temporomandibular joint disorders			x	x
Prior use of a skeletal muscle relaxant*				
skeletal muscle relaxant	in the 30 days prior to the current day of observation	NDCs	x	x
Long-term care residence				
long-term care residence	on current day of observation or in the 30 days prior	long-term care claims	x	x
Therapeutic drug monitoring for warfarin				
international normalized ratio, prothrombin time	on current day of observation or in the 7 days prior	CPT, HCPCS	x	
Average daily warfarin dose				
average daily dispensed dose for warfarin	On current day of observation; set minimum to 0.5 x minimum daily dose per labeled indications; set maximum to 2.0 x maximum daily dose per labeled indications; if less than minimum, set to minimum. If greater than maximum, set to maximum.	calculated as ([dispensed tablet strength] x [dispensed quantity]) / (days' supply)	x	
Average daily skeletal muscle relaxant dose				
average daily dispensed dose for skeletal muscle relaxants	On current day of observation; set minimum to smaller of 0.5 x (minimum daily dose per labeled indications) or (minimum tablet / capsule strength); set maximum to 2.0 x maximum daily dose per labeled indications; if less than minimum, set to minimum. If greater than maximum, set to maximum.	Calculated as ([dispensed tablet strength] x [dispensed quantity]) / (days' supply)	x	

CM = clinical modification; CNS = central nervous system; CPT = Current Procedural Terminology; CYP = cytochrome P450; ICD-9 = International Classification of Diseases 9th revision; G-CSF = granulocyte-colony stimulating factor; GERD = gastroesophageal reflux disease; GM-CSF = granulocyte-macrophage colony-stimulating factor; HCPCS = Healthcare Common Procedure Coding System; HRT = hormone replacement therapy; ICS = inhaled corticosteroid; LMWH = low molecular weight heparin; NDC = National Drug Code; NSAID = nonsteroidal anti-inflammatory drug; IS = ischemic stroke; SNRI = serotonin-norepinephrine reuptake inhibitor; SSRI = selective serotonin reuptake inhibitor; VTE = venous thromboembolism

* Clinical relevance defined by Flockhart DA. Drug Interactions: Cytochrome P450 Drug Interaction Table. Indiana University School of Medicine. https://drug-interactions.medicine.iu.edu.

** Other than that currently being examined

5. Outcomes

Operational definitions[31-36] and quantitative measures of algorithm validity[31,35] are presented in **eMethods Table 3**.

eMethods Table 3. Operational definition of thromboembolism, the composite outcome of interest

Thromboembolism component	Diagnosis descriptor	ICD-9-CM diagnosis code(s)*	Diagnosis position and type / claim type	PPV / sensitivity
venous thromboembolism	iatrogenic pulmonary embolism and infarction	415.11	Principal position inpatient discharge diagnosis / MAX inpatient, short-stay MedPAR, or long-stay MedPAR claim	~95% / ~77%[15]
	other pulmonary embolism and infarction	415.19		
	phlebitis and thrombophlebitis of femoral vein	451.11		
	other phlebitis and thrombophlebitis	451.19		
	phlebitis and thrombophlebitis of lower extremities, unspecified	451.2		
	phlebitis and thrombophlebitis of unspecified site	451.9		
	thrombophlebitis migrans	453.1		
	embolism and thrombosis of inferior vena cava	453.2		
	acute venous embolism and thrombosis of deep vessels of lower extremity	453.4X		
	acute venous embolism and thrombosis of other specified veins	453.8X		
	embolism and thrombosis of unspecified site	453.9		
ischemic stroke	occlusion and stenosis of precerebral arteries**	433.X1		~88% / ~74%[19]
	occlusion of cerebral arteries	434.X†		
	acute, but ill-defined, cerebrovascular disease	436.X		

ICD-9-CM = International Classification of Diseases 9th Revision Clinical Modification; MAX = Medicaid Analytic Extract; MedPAR = Medicare Provider Analysis and Review; PPV = positive predictive value

* X indicates wildcard, i.e., values ranging from 0–9
** excluding mentions of cerebral infarction
† excluding 434.X0

6. Statistical analysis

For each SCCS, we constructed an analytic file in which the unit of analysis was the person-day of observation time. The dependent variable was an indicator for outcome. Independent variables included the subject's observation period, the time window of exposure within the observation period, and time-varying covariates discussed above. The primary analysis compared outcome incidence rates during all exposed time (i.e., agnostic to time window) vs. unexposed time. We used conditional Poisson regression models to estimate IRRs and 95% confidence intervals.[27,37] We conducted numerous secondary analyses (**eMethods Table 4**) to examine the robustness of our findings and assess potential violations of the design's underlying assumptions as a measure of good practice.[38] We calculated ratios of IRRs in which effect estimates for each skeletal muscle relaxant with warfarin and with ICSs were in the numerator and denominator, respectively. The variance of the ratio of IRRs (and corresponding 95% confidence interval) was calculated using the delta method.[39] We conducted analyses using SAS version 9.4 (SAS Institute Inc.: Cary, NC). The University of Pennsylvania's institutional review board approved this research via expedited procedure set forth in 45 CFR 46.110.

eMethods Table 4. Prespecified secondary analyses

Secondary analysis	Rationale
Further elucidating the association between muscle relaxant exposure and outcome	
Stratify exposed time into mutually exclusive periods from initiation of concomitance: 0–15, 16–30, 31–60, 61–120, and 121–180 days.	Facilitates elucidation of duration-response effects
Stratify exposed time by clinically meaningful dose cut points for muscle relaxant	Facilitates elucidation of dose-response effects
Deconstruct composite outcome and separately examine VTE and IS	Facilitates elucidation of differential effects by outcome component
Examine order of initiation of concomitance*	Facilitates examination of time periods during which an interaction would be biologically plausible
Assessing SCCS underlying assumptions, minimizing the role of bias and/or confounding	
Exclude observation time preceding the initial exposed period (i.e., conduct left-censored unidirectional SCCS study)	Minimizes reverse causality bias, although may be prone to exposure-trend bias
Exclude observation time following the end of the initial exposed period (i.e., conduct right-censored unidirectional SCCS study)	Minimizes reverse causality bias, although may be prone to exposure-trend bias
Exclude subjects with >1 outcome during the observation period	Assesses impact of potential failure of outcome independence assumption; second or later outcomes, if influenced by initial outcome, may cause failure of underlying Poisson model
Exclude subjects that die during the observation period	Alleviates concern for death as an ultimate interferent event (and thereby censoring observation time), especially since the outcome of interest increases mortality; alleviates concern for immortal time bias
Increase grace period length to approximate 70% adherence	Assesses sensitivity of findings to assumptions about potential exposure misclassification
Decrease grace period length to approximate 90% adherence	

IS = ischemic stroke; SCCS = self-controlled case series; VTE = venous thromboembolism

* Hennessy et al. *Clin Pharmacol Ther* 2016;99(1):92-100.

SUPPLEMENTAL MATERIAL | Tables

Table S1: Rationale for answering this study question in a Medicaid population

Rationale	Reference
Vulnerable population, with high prevalence of multiple chronic conditions, that is at greater baseline risk for adverse drug events	Office of Disease Prevention and Health Promotion. National Action Plan for Adverse Drug Event Prevention. 2014.
Large proportions of women, minorities, and persons socioeconomically disadvantaged—populations poorly represented in clinical trials	Mosenifar Z. Population issues in clinical trials. *Proc Am Thorac Soc* 2007;4(2):185-187.
Benefit covers 20% of the United States population	Nam et al. Nonsteroidal anti-inflammatory drug choice and adverse outcomes in clopidogrel users: A retrospective cohort study. *PLoS One* 2018;13(3):e0193800.
Large number of muscle relaxant users	Centers for Disease Control and Prevention. National Center for Health Statistics. National Ambulatory Medical Care Survey, 2016. 2019.
Independent of population in which putative drug interaction signals were generated	Zhou M. Identifying signals of potential drug-drug interactions involving oral anticoagulants using real world clinical data. 2019. Publicly Accessible Penn Dissertations. 3445. Available at: https://repository.upenn.edu/edissertations/3445.

Table S2: Adjusted incidence rate ratios and ratios of incidence rate ratios for thromboembolism, for prespecified primary analyses

	Object drug of interest: Warfarin			Negative control object drug: ICS			Ratio of warfarin to ICS		
Muscle relaxant precipitant drug	adjusted IRR	95% confidence interval		adjusted IRR	95% confidence interval		adjusted rIRR	95% confidence interval	
Baclofen	0.81	0.53	1.25	0.77	0.40	1.51	1.05	0.47	2.33
Carisoprodol	0.85	0.53	1.39	**1.99**	1.04	3.83	**0.43**	0.19	0.97
Cyclobenzaprine	0.95	0.69	1.32	1.13	0.71	1.80	0.85	0.48	1.50
Metaxalone	***0.31**	0.13	0.77	1.50	0.38	5.95	0.21	0.04	1.08
Methocarbamol	0.84	0.44	1.63	1.50	0.56	4.00	0.56	0.17	1.83
Tizanidine	****3.44**	1.53	7.78	0.39	0.10	1.49	**8.83**	1.84	42.5

ICS = inhaled corticosteroid; INR = international normalized ratio; IRR = incidence rate ratio; rIRR = ratio of incidence rate ratios

Bolded effect estimates met the traditional threshold for statistical significance.

* Statistically significant protective IRR consistent with potential INR elevation (as large as +1.0 units) reported in Zhou M. Identifying signals of potential drug-drug interactions involving oral anticoagulants using real world clinical data. 2019. Publicly Accessible Penn Dissertations. 3445.

** Statistically significant elevated IRR consistent with potential INR reduction (as large as -0.4 units) reported in Zhou M. Identifying signals of potential drug-drug interactions involving oral anticoagulants using real world clinical data. 2019. Publicly Accessible Penn Dissertations. 3445.

Table S3: Adjusted incidence rate ratios for thromboembolism, for prespecified secondary analyses

Prespecified secondary analysis	Incidence rate ratios (95% confidence interval), *warfarin + muscle relaxant* vs. *warfarin alone*					
Further elucidating the association between precipitant drug exposure and outcome						
Stratify exposed time into mutually exclusive periods from initiation of concomitance: 0–15, 16–30, 31–60, 61–120, and 121–180 days.		0-15d	16-30d	31-60d	61-120d	121-180d
	baclofen	0.83 (0.53-1.32)	0.79 (0.44-1.42)	1.06 (0.53-2.13)	0.28 (0.08-1.04)	0.29 (0.03-2.46)
	carisoprodol	0.92 (0.56-1.51)	0.87 (0.46-1.64)	0.71 (0.32-1.59)	0.43 (0.12-1.50)	0.34 (0.03-3.30)
	cyclobenzaprine	0.92 (0.65-1.32)	1.36 (0.82-2.25)	0.91 (0.46-1.80)	1.47 (0.53-4.07)	ND
	metaxalone	**0.25** (0.09-0.72)	0.79 (0.25-2.49)	0.47 (0.06-3.59)	ND	ND
	methocarbamol	0.82 (0.41-1.66)	0.96 (0.33-2.80)	0.81 (0.15-4.27)	1.16 (0.18-7.46)	ND
	tizanidine	**4.58** (2.00-10.48)	2.28 (0.54-9.67)	1.88 (0.35-10.19)	0.76 (0.08-7.41)	ND
Stratify exposed time by daily dose cut points for muscle relaxant		≤ median average daily dose* for given muscle relaxant**	> median average daily dose* for given muscle relaxant**			
	baclofen	0.77 (0.47-1.26)	0.93 (0.42-2.03)			
	carisoprodol	0.66 (0.37-1.17)	1.53 (0.66-3.59)			
	cyclobenzaprine	1.04 (0.68-1.60)	0.85 (0.52-1.38)			
	metaxalone	0.54 (0.14-2.18)	**0.23** (0.07-0.72)			
	methocarbamol	**2.27** (1.01-5.10)	**0.20** (0.07-0.60)			
	tizanidine	**4.11** (1.24-13.65)	**3.00** (1.04-8.70)			
Deconstruct composite outcome and separately examine VTE and IS		VTE	IS			
	baclofen	1.30 (0.78-2.17)	**0.23** (0.09-0.57)			
	carisoprodol	0.89 (0.53-1.50)	1.73 (0.39-7.65)			
	cyclobenzaprine	0.97 (0.66-1.40)	1.06 (0.49-2.31)			
	metaxalone	**0.33** (0.13-0.87)	ND			
	methocarbamol	0.74 (0.37-1.49)	0.94 (0.14-6.49)			
	tizanidine	**2.91** (1.21-6.97)	1.28 (0.10-16.61)			
Examine order of initiation of concomitance		Object-triggered	Precip.-triggered	Synchrony-triggered		
	baclofen	0.78 (0.42-1.47)	0.77 (0.47-1.26)	2.77 (0.53-14.47)		
	carisoprodol	1.35 (0.67-2.70)	0.62 (0.34-1.12)	ND		
	cyclobenzaprine	1.59 (0.95-2.67)	0.72 (0.47-1.11)	0.56 (0.12-2.58)		
	metaxalone	0.35 (0.10-1.22)	**0.29** (0.09-0.98)	ND		
	methocarbamol	1.57 (0.56-4.38)	0.46 (0.17-1.22)	1.54 (0.21-11.07)		
	tizanidine	**6.85** (1.86-25.17)	1.00 (0.30-3.38)	ND		
Assessing SCCS underlying assumptions, minimizing the role of bias and/or confounding						
Exclude observation time preceding the initial exposed period (i.e., conduct left-censored unidirectional SCCS study)	baclofen	1.12 (0.65-1.95)				
	carisoprodol	1.20 (0.64-2.25)				
	cyclobenzaprine	0.94 (0.64-1.38)				
	metaxalone	0.41 (0.13-1.25)				
	methocarbamol	0.82 (0.38-1.77)				
	tizanidine	**7.35** (2.50-21.64)				
Exclude observation time following the end of the initial exposed period (i.e., conduct right-censored unidirectional SCCS study)	baclofen	0.68 (0.43-1.08)				
	carisoprodol	0.85 (0.49-1.46)				
	cyclobenzaprine	0.92 (0.63-1.33)				
	metaxalone	**0.21** (0.07-0.60)				
	methocarbamol	0.89 (0.44-1.78)				
	tizanidine	**3.22** (1.31-7.94)				
Exclude subjects with >1 outcome during the observation period	baclofen	**0.56** (0.35-0.92)				
	carisoprodol	0.69 (0.38-1.24)				
	cyclobenzaprine	1.20 (0.82-1.75)				
	metaxalone	0.39 (0.15-1.02)				
	methocarbamol	0.79 (0.40-1.56)				
	tizanidine	**3.07** (1.24-7.60)				
Exclude subjects that die during the observation period	baclofen	0.87 (0.56-1.34)				
	carisoprodol	0.91 (0.55-1.48)				
	cyclobenzaprine	0.98 (0.70-1.36)				

	metaxalone	**0.30** (0.11-0.77)
	methocarbamol	0.84 (0.44-1.63)
	tizanidine	**3.42** (1.51-7.74)
Increase grace period length to approximate 70% adherence	baclofen	0.75 (0.50-1.13)
	carisoprodol	0.85 (0.55-1.33)
	cyclobenzaprine	1.01 (0.74-1.38)
	metaxalone	**0.28** (0.12-0.66)
	methocarbamol	0.84 (0.46-1.54)
	tizanidine	**3.69** (1.71-7.97)
Decrease grace period length to approximate 90% adherence	baclofen	0.81 (0.51-1.29)
	carisoprodol	0.68 (0.41-1.14)
	cyclobenzaprine	0.93 (0.65-1.33)
	metaxalone	**0.32** (0.12-0.81)
	methocarbamol	0.68 (0.33-1.37)
	tizanidine	**2.78** (1.21-6.43)

IS = ischemic stroke; ND = not detectable (or lower bound of 95% confidence interval <0.01); SCCS = self-controlled case series; VTE = venous thromboembolism

Incidence rate ratios meeting the traditional threshold for statistical significance have been bolded.

* Median average daily doses were: baclofen (30 milligrams [mg]), carisoprodol (1050 mg), cyclobenzaprine (20 mg), metaxalone (1600 mg), methocarbamol (2000 mg), and tizanidine (8 mg)

** Versus muscle relaxant unexposed

SUPPLEMENTAL MATERIAL | Figure

Figure S1: Persons under study by object drug, by calendar year

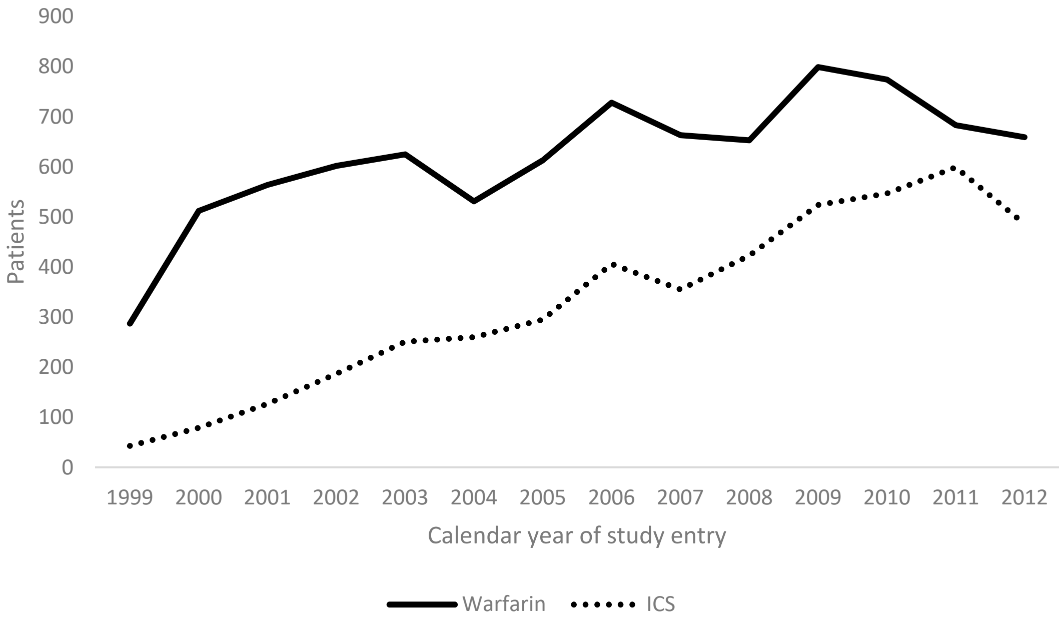

Supplement: Supplementary file 1 [file medicina-58-01171-s001.zip › medicina-1841420-supplementary.pdf]
